# Cervical Squamous Cell Carcinoma Diagnosis by FTIR Microspectroscopy

**DOI:** 10.3390/molecules29050922

**Published:** 2024-02-20

**Authors:** Maria M. Félix, Mariana V. Tavares, Inês P. Santos, Ana L. M. Batista de Carvalho, Luís A. E. Batista de Carvalho, Maria Paula M. Marques

**Affiliations:** 1 Molecular Physical-Chemistry R&D Unit, Department of Chemistry, University of Coimbra, 3004-535 Coimbra, Portugal; mmfelix@uc.pt (M.M.F.); mariana.vide.tavares@ipoporto.min-saude.pt (M.V.T.); ips@uc.pt (I.P.S.); pmc@ci.uc.pt (M.P.M.M.); 2 Gynaecology Department, Portuguese Oncology Institute of Porto, 4200-072 Porto, Portugal; 3 Department of Life Sciences, Faculty of Science and Technology, University of Coimbra, 3000-456 Coimbra, Portugal

**Keywords:** human cervical cancer, squamous cell carcinoma, cryopreserved cervical tissue, FTIR microspectroscopy, SVM

## Abstract

Cervical cancer was considered the fourth most common cancer worldwide in 2020. In order to reduce mortality, an early diagnosis of the tumor is required. Currently, this type of cancer occurs mostly in developing countries due to the lack of vaccination and screening against the Human Papillomavirus. Thus, there is an urgent clinical need for new methods aiming at a reliable screening and an early diagnosis of precancerous and cancerous cervical lesions. Vibrational spectroscopy has provided very good results regarding the diagnosis of various tumors, particularly using Fourier transform infrared microspectroscopy, which has proved to be a promising complement to the currently used histopathological methods of cancer diagnosis. This spectroscopic technique was applied to the analysis of cryopreserved human cervical tissue samples, both squamous cell carcinoma (SCC) and non-cancer samples. A dedicated Support Vector Machine classification model was constructed in order to categorize the samples into either normal or malignant and was subsequently validated by cross-validation, with an accuracy higher than 90%.

## 1. Introduction

Cervical cancer (CC) is the fourth most incident cancer, globally. The World Health Organization estimated that 604,000 women were diagnosed with CC and 342,000 patients died from it in 2020 [1]. Most of the cases occur in developing countries, with a higher prevalence in the African Sub-Saharan region and Asia [2,3]. These countries lack screening tests for SCC and have not yet included the vaccine against Human Papillomavirus (HPV) in their National Vaccination Programs (NVPs), which results in the emergence of new cases of cervical tumors [4]. Women from developed countries, on the other hand, are offered vaccines against HPV, which might lead to a decreasing incidence of dysplastic and consequently neoplastic lesions [5,6,7].

The association between cervical cancer and HPV infection is well established [8]. Cervical cancer is primarily caused by the long-lasting infection of certain HPV strains, mainly HPV-16 and HPV-18 [9]. Progression of CC from HPV infection, leading to the onset of high-grade lesions and carcinoma, occurs gradually and may take several years [10]. Thus, an early detection of premalignant lesions is essential to prevent progression to carcinoma [11]. The predominant histopathological subtypes of cervical cancer are squamous cell carcinoma (SCC) and adenocarcinoma (AC), with incidences of 75% and 20%, respectively [12]. Squamous cell carcinoma originates from the thin, flat squamous cells found in the epithelial tissue of the cervix [13].

Currently, the screening methods for these types of tumors are performed through the cytopathological Papanicolaou (Pap) Smear test which has a high specificity (>90%), although the sensibility varies from 74 to 98%; High Risk-Human Papillomavirus (HPV-HR) test, with 97–99% sensibility and 84–89% specificity; and colposcopy, which has 90% sensibility and 35–50% specificity [14,15,16,17]. However, there is apprehension regarding the precision of the aforementioned methods and their subsequent efficacy in facilitating precise screening and early diagnosis of cervix cancer [18]. Currently, the gold standard procedure in cases of abnormal cytology and/or HPV positive results is colposcopy-guided biopsy. Still, this technique requires an experienced operator, and the inter-colposcopist variability in biopsy site selection is a major limitation on the efficiency of the diagnosis, leading to high rates (>30%) of misclassification [19,20]. Therefore, an increasing clinical need arises for more reliable and accurate diagnostic techniques that are as sensitive and specific as possible. Research in optical techniques has been ongoing with a view to address the limitations associated with conventional histopathological diagnosis techniques [21]. Among these, vibrational spectroscopy has emerged as a promising method, with the potential to enhance accuracy and efficiency in cancer diagnosis [22,23,24,25].

In fact, various authors reported the use of vibrational spectroscopy techniques to discriminate normal from malignant tissues. Their findings reveal high rates of sensitivity and specificity, through the application of models employing Support Vector Machine (SVM) and cross-validation [26,27]. Norbert Bergner et al. [28] achieved a significant success in identifying primary tumors and tumor margins using Fourier transform infrared (FTIR) microspectroscopy coupled to SVM, achieving an accuracy above 90%.

FTIR microspectroscopy stands out as the preferred method for early diagnosis of cervical cancer due to its non-invasiveness, high accuracy, and reproducibility. These characteristics facilitate the identification of subtle changes in the chemical profile of highly heterogeneous biological samples, such as cells, tissues, or biofluids, particularly regarding proteins, lipids, carbohydrates, and nucleic acid composition. Moreover, its specificity exceeds that obtained by histopathological analysis, which can only identify morphological changes that occur upon chemical variations. In sum, FTIR microspectroscopy enables a rapid and reliable detection of differences in chemical composition between normal and malignant samples [29,30], which are responsible for subsequent morphological variations observed in histopathology. In the last few years, infrared microspectroscopy has been successfully applied to the diagnosis of various types of malignancies, including lung, breast, thyroid, brain, and skin cancers [31,32,33,34,35,36,37].

The main goal of this study is to use FTIR microspectroscopy to analyze cryopreserved human cervical tissue samples, previously characterized by histopathology (as either normal or malignant (SCC)), aiming to increase the reliability of tumor detection [37,38,39]. FTIR microspectroscopy was combined with the conventional methods (Pap Smear, HPV-HR, and colposcopy) for an early and reliable diagnosis of cervical cancer. The FTIR bands reflecting the chemical differences between healthy and malignant tissues were identified as spectral biomarkers, and a classification model based on this spectroscopic data was built to categorize the samples in either normal or SCC groups. The model was then validated using a cross-validation procedure.

## 2. Results and Discussion

The human cervical tissues, cryopreserved at −80 °C, allowed an analysis of the specimens as close as possible to the natural state since their properties were kept by freezing, without the need for preservatives, allowing reliable FTIR microspectroscopy results to be obtained. This represents a great benefit compared to similar studies in the area which often use preservatives, fixatives, or dyes, which impact the original state of the sample components (e.g., protein conformation), apart from often yielding noticeable bands that may hinder an accurate detection of the signals from the biological sample [40,41,42,43]. For instance, paraffin, often employed in the preparation of tissue specimen for analysis, can affect the morphology of the tissue, yielding blended spectra from which it is impossible to draw reliable conclusions. In addition, crucial spectral regions of the biological sample may be overruled by the paraffin signals [41,44,45]. Hence, some studies involving paraffin-embedded samples use dewaxing procedures, in order to achieve spectra that are as close as possible to the tissue’s natural state, by one of two methods: (i) chemically, by washing the sample with organic solvents (e.g., xylene and ethanol), which implies that some solvent residues may remain in the sample or that parts of the tissue are lost during the process [27]; or (ii) digitally, by subtracting the paraffin, which may introduce digital artifacts into the spectra.

In this study, a total of 37 fresh cryopreserved cervical tissue samples were examined; 12 were malignant tissues extracted from women with cervical SCC tumors, while the remaining 25 samples, from normal cervical tissue, were used as controls. The malignant samples are part of the study group and were obtained through biopsy of mainly SCC ulcerated lesions, mostly from Caucasian and unvaccinated women. The control samples were sourced from Caucasian women, who underwent hysterectomy due to gynecologic tumors other than cervical cancer (ovarian cancer, uterine cancer, vaginal cancer, etc.), or due to an increased risk mutation for gynecological tumors. They were categorized to the control group after histopathological confirmation of the absence of cancer cells in the cervical tissue.

Chemical insights at the molecular level were obtained from the cervical tissue samples through FTIR microspectroscopy analysis, comparing normal and malignant SCC specimens. While cancerous specimens could be analyzed in their totality, as they exclusively contained invasive malignant cells, the control samples were probed by FTIR solely in the epithelium, since SCC originates in the epithelium and a chemical characterization of the normal tissue in this area is therefore required in order to compare with the malignant tissue [46].

**Figure 1:**
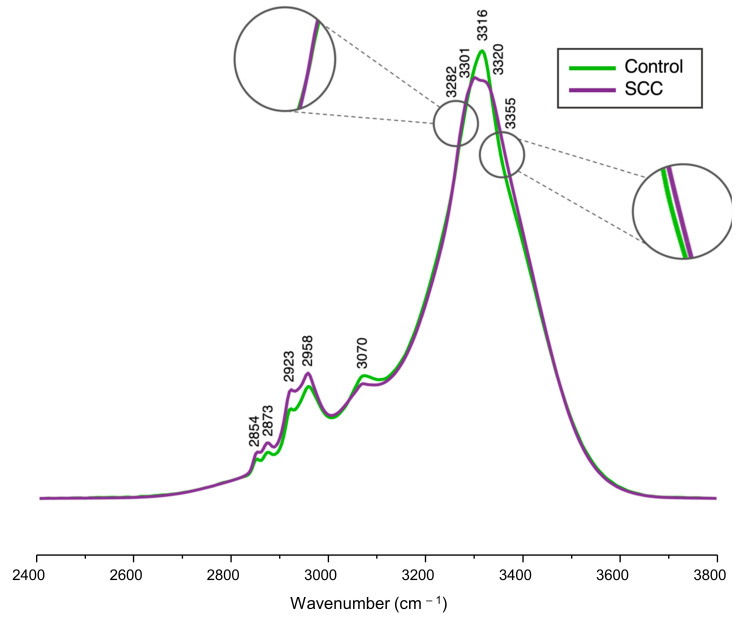
Average FTIR spectra (2400–3800 cm^−1^) of cryopreserved normal and malignant human cervical tissue samples.

The fingerprint region of the infrared spectrum (600–1800 cm^−1^) was inaccessible for analysis since the samples were measured on glass slides, which are commonly used in the clinics due to their affordability and compatibility with histological techniques. However, this substrate produces intense infrared bands in the 600–1800 cm^−1^ spectral range (e.g., δ(Si-O) and δ(B-O) at 500–1200 cm^−1^, and ν(Si-O-Si) at 1200–1500 cm^−1^) [47]. Thus, only the high-wavenumber range (2400–3800 cm^−1^) could be accessed in the present study. Figure 1 shows the average FTIR spectra for both normal and malignant cervix tissue samples, as well as the corresponding spectral assignments (detailed in Table 1).

**Figure 2:**
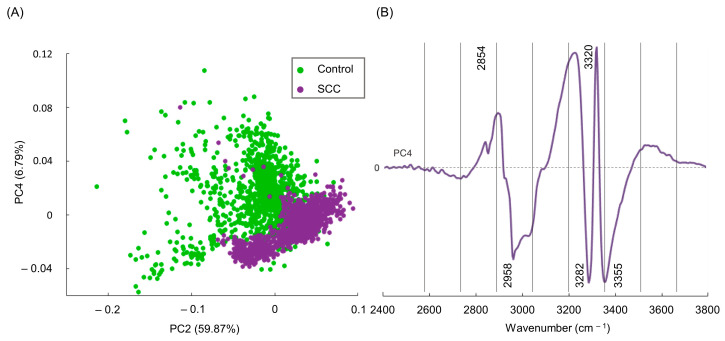
(**A**) PCA score of FTIR data (PC2 vs. PC4) for cryopreserved normal vs. SCC human cervical tissue and (**B**) respective PC4 loading plot of the FTIR high-wavenumber region.

These average FTIR spectra provided a visual characterization of the chemical composition for the samples under analysis, revealing main spectral changes between the control (normal cervical tissue) and the study (cervical SCC) groups. The following bands exhibited the most significant variations, displaying a higher intensity in SCC when compared to the control: ν_s_(CH_2_) at 2854 cm^−1^, ν(CH) at 2873 cm^−1^, ν_as_(CH_2_) at 2923 cm^−1^, ν_as_(CH_3_) at 2958 cm^−1^, and Amide A at 3355 cm^−1^. The feature at 3282 cm^−1^, from Amide A, exhibited a small inflection (Figure 1) that caused a negative loading upon principal component analysis (PCA) (Figure 2B), contributing more notably to the carcinogenic samples. The signals at 3301 cm^−1^ and 3320 cm^−1^ observed in the tumoral samples indicate a splitting of the band at 3316 cm^−1^ identified in the mean spectra of the normal tissues. A signal at 3320 cm^−1^ in the PC4 loading, ascribed to ν(OH), is proposed to result from intensity variations between the average spectra of the control versus SCC samples. At 3355 cm^−1^ (Amide A), an inflection was observed, resulting in a loading that contributed mainly for the cancerous samples, as evidenced by the PCA analysis of the spectra (Figure 2).

In order to obtain accurate results, FTIR data must be processed through multivariate analysis (MVA), enabling a more detailed information regarding the measured spectra, impossible to attain by simple spectral observation. In fact, MVA enables the unveiling of even the subtlest chemical differences between samples, which will lead to the diagnosis [48]. Additionally, some research groups use automated sample analysis, developed with deep-learning algorithms trained to recognize patterns. These efforts aim to achieve classification models to predict the category of the sample of the same type of tissue, enhancing the precision of decision-making processes regarding the diagnosis of cervical cancer [27,48]. To ensure that the observed spectral changes reflect real differences between normal and SCC samples, PCA was performed to reduce the dimensionality of the dataset and to serve as an input for further SVM model classification procedures. When performing PCA, the score plots of the high-wavenumber region were analyzed, and revealed that PC1, PC2, and PC3 failed to discriminate normal from malignant tissue samples (Figure S1, Supplementary Material). Nevertheless, a separation between these groups was accomplished through the analysis of PC4 (accounting for 6.79% of the data variance), identified as the component that better distinguished the two types of specimens (Figure 2A). Although PC4 accounted solely for 6.79% of the data variance, this is believed to be an accurate result as cancerous tissue originates from normal tissue, implying significant similarities between the normal and tumoral samples.

Upon examining the PCA score distribution for the spectral data presently gathered (Figure 2A), it becomes evident that PC2 fails to discriminate between the two classes—SCC and normal tissues. However, when observing the score distribution along the *y*-axis (PC4), clear differences emerge between both specimens. Scores above zero in the *y*-axis consist mostly of control samples, whereas those below zero are mainly from malignant tissues (although overlapped with some non-malignant ones). Thus, the loading of PC4 enabled the unveiling of the chemical differences between the two categories. Loadings above zero in the PC4 plot mostly contributed to the control group, while loadings below zero were more indicative of malignant samples (Figure 2B).

The PC4 loading plot revealed several wavenumbers deemed as reliable spectral biomarkers (e.g., ν(CH_3_), Amide A ν(NH)), associated with significant biochemical variations between the two classes (Figure 2B). More specifically, bands assigned to ν_s_(CH_2_) at 2854 cm^−1^ and ν(OH) at 3320 cm^−1^ were considered as spectral biomarkers for the normal samples. Conversely, SCC samples exhibited a lower intensity for these bands, due to decreased contributions from CH_2_ and OH groups found in proteins, lipids, and carbohydrates. This reduction is attributed to biochemical alterations occurring during the development of SCC. In turn, signals from ν_as_(CH_3_), at 2958 cm^−1^, and Amide A (ν(NH)), at 3282 and 3355 cm^−1^, were more intense in cancer samples as compared to the controls. In sum, these infrared bands can be consistently used as spectral biomarkers of cervical cancer. These infrared variations can also be partly ascribed to changes in lipid composition within neoplastic cells. This alteration in lipid composition is essential for supporting tumor proliferation and facilitating a more rapid lipid signaling function, which regulates various cellular processes. Consequently, any disruption of these processes may lead to cancer development and/or progression [49,50,51,52]. Additionally, modifications in the ν_as_(CH_3_) vibrational mode of proteins may indicate alterations in protein structure and side chain conformation, or even changes in the microenvironment around the proteins associated with tumoral progression [27,53]. Variations in the Amide A signal (ν(NH)) can be linked to protein carcinogenesis-induced conformational changes, particularly associated with reorganization of the hydrogen bond patterns, altered expression, and abnormal synthesis of specific proteins and post-translational modifications [54]. Changes in the methyl CH stretching and Amide A modes have been documented for skin and breast cancer [27,33,55], which corroborates the current results.

**Figure 3:**
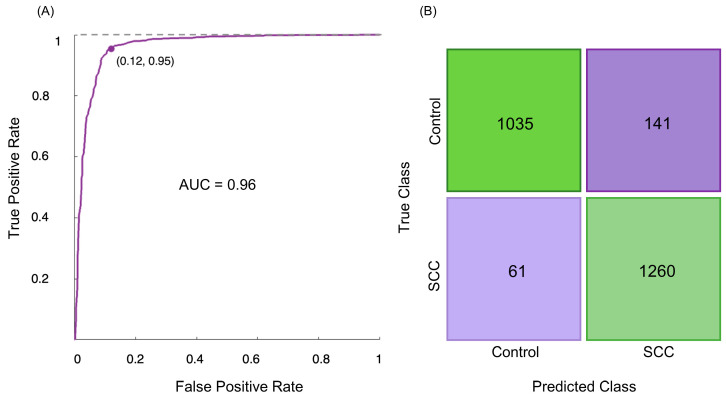
(**A**) ROC curve of discrimination between human cervical normal and SCC specimens, using the SVM classification model on the FTIR data; (**B**) confusion matrix of the FTIR classification model.

Upon determination of the principal component that effectively differentiated between normal and cervical SCC groups, along with the identification of spectral bands that can be considered biomarkers of cervical cancer, a classification model was constructed to classify the samples based on the reduced data from the PCA. The FTIR classifiers were developed using a SVM procedure, which is a supervised machine learning algorithm applied to classify the samples based on a 5-fold cross-validation method [56,57]. Upon using the classification learner generated by MATLAB, a receiver operating characteristic (ROC) curve (Figure 3A) was constructed at different discriminating threshold levels, showing the performance of the classification model created to distinguish cervical SCC from normal tissue. The corresponding ROC curve displayed the performance of the classification model with an area under the curve (AUC) of 0.96, where an AUC of 1.0 corresponds to a perfect classification test.

The accuracy of the FTIR method applied in this study is demonstrated by the cross-validation confusion matrix (Figure 3B), which compares the FTIR analysis to the gold-standard histopathological diagnosis. The confusion matrix demonstrated that among 1176 spectra from the control group, 141 (12.0%) were misclassified as belonging to the SCC group, signifying that 1035 spectra were correctly classified as normal (Figure 3B). On the other hand, 1260 out of 1321 spectra of malignant samples (93.4%) were accurately classified as belonging to the SCC study group, which means that 61 malignant specimens were incorrectly classified as normal (Figure 3B). Hence, the malignant samples were discriminated from the normal ones with 94% sensitivity (identification of true positives), 90% specificity (identification of true negatives), and 92% accuracy.

The present study constitutes a significant advancement in human cervical cancer diagnosis. Through the application of FTIR microspectroscopy in conjunction with traditional histological methods, pathologists can attain an earlier, more reliable, and accurate diagnosis. This is facilitated by FTIR’s capacity to provide detailed chemical profiles of biological and highly heterogeneous samples, allowing the detection of even subtle chemical disparities, namely between malignant and normal tissues with very high accuracy, specificity, and sensitivity. This method identifies chemical variations prior to the emergence of morphological changes that can be detected by histopathology. Therefore, this is a promising approach for an early diagnosis of cancer, leading to improved cancer survival rates. It should not be forgotten that delayed diagnosis remains the primary cause of death from cervical cancer.

This study has effectively addressed a significant challenge regarding the preservation conditions of the tissue samples intended for spectroscopic analysis, showing that this type of analysis is possible for frozen tissue (unfixed and unpreserved). This procedure involved immediate cryopreservation of the freshly excised tissue samples, without any additional handling, yielding FTIR data that exclusively reflects the molecular composition of tissue in its native state, either normal or diseased.

Although only the FTIR high-wavenumber region was measured, its analysis provided sufficient information regarding the biochemical characteristics of the tissue types, enabling an accurate discrimination between them. The primary changes observed between groups were identified in lipids and proteins—ν(CH_3_) and Amide A playing a key role in differentiating tumoral from normal samples. These molecular alterations are also corroborated by several reported studies on tissue diagnosis using FTIR spectroscopy [27,33,58].

The FTIR technique is considered the most suitable vibrational spectroscopic method for translation to the clinical workflow, since it is faster when compared to Raman microspectroscopy and its spectral quality is not dependent on focus, unlike Raman. Upon application of the SVM model to the PCA data, very promising results were achieved regarding the sample classification, attaining an accuracy over 90%.

However, despite these results, certain limitations were identified, namely the use of glass slides as substrates, which restricts spectral acquisition to the high-wavenumber region. Note that this option was taken to maintain sample conditions that were as close as possible to those used in clinical centers for histopathological analysis, thus minimizing disruptions to their standard workflow. Some studies have used calcium fluoride crystal slides in order to obtain information across the entire spectral range (both fingerprint and high wavenumber); however, these are rather more expensive than glass and are not suitable for application in routine clinical diagnosis [59,60].

## 3. Materials and Methods

### 3.1. Sample Preparation

The samples used in this study were sourced from cervical tissue obtained from patients followed at the Gynaecology Department of the Portuguese Oncology Institute—Porto (IPO—Porto).

Normal cervical tissue samples (n = 25) were obtained from 25 patients undergoing hysterotomy due to either malignant gynecological tumors or for prophylactic purposes in individuals with an increased risk of gynecological tumors (such as Lynch syndrome). Carcinogenic tissue specimens (n = 12) from 12 patients were collected through biopsy. All samples were provided by IPO—Porto after patient’s informed consent and ethical approval according to the research protocol reviewed and approved by the Ethical Committee Board of IPO—Porto (according to the Helsinki Declaration Informed consent).

Upon excision, the tissue was promptly cryopreserved at −80 °C, without additional preparation. Subsequently, two contiguous sections of tissue were cryostat cut and mounted on glass slides: a 10 μm thick, unstained non-paraffinized section for FTIR analysis and a 3 μm thick hematoxylin and eosin (H&E)-stained section for histopathological analysis (for comparative purposes). This approach offers an advantage over paraffinized tissues, as the corresponding infrared spectra are free from the interference of paraffin signals.

In order to ensure preservation of the samples during their transport from IPO—Porto to the Molecular Physical Chemistry R&D Unit of the University of Coimbra, the slides were carefully packed in dry ice to prevent thawing and tissue degradation. Upon arrival, the slides were promptly transferred to the −80 °C freezer for storage until spectroscopic analysis, thereby ensuring sample integrity and quality.

For the FTIR measurements, the tissue samples were retrieved from the −80 °C freezer and washed with a saline solution to remove the remaining blood, which would interfere with the spectroscopic results due to the presence of high amounts of hemoglobin [61].

### 3.2. Data Acquisition

FTIR microspectroscopy acquisition was performed using a Bruker Hyperion 2000 microscope (Bruker Optik GmbH, Ettlingen, Germany) with a liquid nitrogen cooled Mercury-Cadmium-Telluride (MCT) detector, in transmission mode, coupled to a Bruker Optics Vertex 70 spectrometer (Bruker Optik GmbH, Ettlingen, Germany) equipped with a Ge on KBr substrate beam splitter, both purged by CO_2_-free dry air. Each acquisition was performed with 4 cm^−1^ resolution and 64 scans using a 15× Cassegrain for both condenser and objective. The background was measured every 10 spectra. All data acquisition was performed using OPUS 9.1 software (Bruker Optik GmbH, Ettlingen, Germany). A 3-term Blackman–Harris apodization function was applied. Infrared transmission spectra were obtained by rationing to a background measured from a clean area of the sample substrate (where no tissue was present).

**Figure 4:**
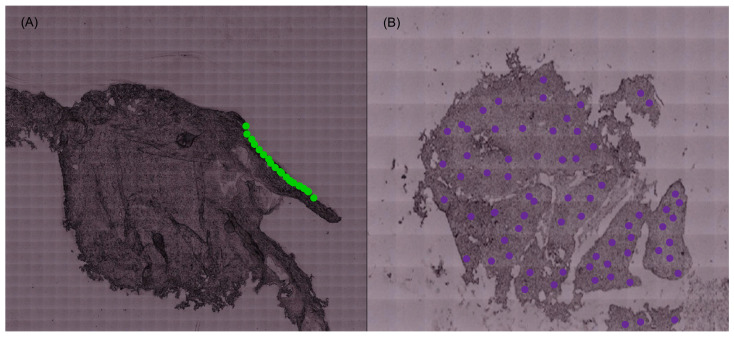
(**A**) Spectra obtained in the epithelium of the control samples. (**B**) Spectra obtained for the totality of the malignant samples (illustrative figure; the points do not correspond to the spectra acquired for the tissue samples).

The spectra of the normal cervical tissue samples were acquired solely in the epithelium, while the spectra of the malignant samples were obtained for the totality of the sample (as schematically illustrated in Figure 4).

### 3.3. Data Processing

The raw spectral data were obtained from the FTIR analysis of the normal and malignant tissue samples.

Resonance Mie scattering correction was applied to the FTIR data through the ESMC algorithm, to compensate for scattering effects from the surface of the samples. Since only the high-wavenumber region was probed, the spectra were noise filtered using PCA (25 PCs were kept for data reconstruction). Normalization (standardizing the area under the curve to the group median) was performed. Outliers were excluded while the rest of the dataset was passed on for modelling.

### 3.4. Data Analysis and Machine Learning

Unsupervised PCA was used to reduce the dimensionality of the dataset and to perform an exploratory analysis in order to discern patterns within the spectral data. The sequence of the principal components (PCs) reflects their significance within the dataset, with PC1 representing the greatest source of variation observed.

Subsequently, a classification model was built combining PCA and SVM, to discriminate the samples between malignant and normal categories. In the PCA-SVM model creation, PCA was found to feed the most relevant features (reduced-dimension data), while SVM learns data patterns between groups of observations with the aim of finding the hyperplane that best separates the classes (through the chosen kernel function), and classifies the samples, distinguishing one class from another by analyzing the patterns of information associated with those observations [57]. Combining PCA and SVM is particularly useful when dealing with this type of spectral data, due to its high variable dimensionality, and ensures the model robustness.

The linear kernel function was selected in order to avoid overfitting, as well as to obtain reliable classification results [62]. The created model was evaluated with a 5-fold cross-validation approach. The model’s accuracy and diagnosis capacity were assessed based on the values for sensitivity, specificity, accuracy, and area under the curve (AUC) of the receiver operating characteristic (ROC) curve (an AUC = 1 indicating a perfect classification). All computations were performed using MATLAB_R2023b (MathWorks, Natick, MA, USA).

## 4. Conclusions

This work aimed to apply FTIR microspectroscopy as a diagnostic tool, coupled with the already existing histopathological methods, with the aim of improving human cervical cancer diagnosis. Current limitations in clinically used methods for cervical cancer screening and diagnosis undermine the efficacy of chemotherapy and reduce the patient´s life expectancy, particularly in developing countries, where cervical SCC has a higher incidence rate. Given the severity of this disease, achieving an early and reliable diagnosis is imperative.

Human cervical tissue samples were probed by FTIR microspectroscopy, alongside control (non-malignant) specimens. Distinct vibrational spectroscopic signatures were obtained for each group of samples, enabling a precise identification of the biochemical changes associated with cervical SCC. Upon chemometric analysis (PCA), several spectral biomarkers of cervical cancer were identified—ν_as_(CH_3_) and Amide A (ν(NH)) were considered—thus enabling a reliable differentiation between SCC and normal samples.

FTIR microspectroscopy was shown to be a promising tool to provide pathologists with detailed and consistent chemical information on human cervical squamous cell carcinoma. This approach can significantly enhance our understanding of the molecular alterations that prompt tissue adaptability in neoplastic progression, which, in turn, may enable an early detection of high-risk cervical cancer, ultimately improving its clinical outcome.

## Figures and Tables

**Table 1:** FTIR assignment (high-wavenumber region) for cryopreserved normal and malignant human cervical tissue samples.

Wavenumber (cm^−1^)	Proteins	Lipids	Carbohydrates
2854	ν_s_(CH_2_)	ν_s_(CH_2_)	ν_s_(CH_2_)
2873		ν(CH)	ν(CH)
2923	ν_as_(CH_2_)	ν_as_(CH_2_)	ν_as_(CH_2_)
2958	ν_as_(CH_3_)	ν_as_(CH_3_)	ν_as_(CH_3_)
3282	Amide A; ν(NH)		
3070	Amide B		
3301	Amide A	ν(OH)	ν(OH)
3316		ν(OH)	ν(OH)
3320		ν(OH)	ν(OH)
3355	Amide A		

ν_s_—symmetric stretching; ν_as_—anti-symmetric stretching.

## Data Availability

The data presented in this study are available on request from the corresponding author.

## Supplementary Materials

The following supporting information can be downloaded at: https://www.mdpi.com/article/10.3390/molecules29050922/s1.
