# Supplementary material for: Cervical Squamous Cell Carcinoma Diagnosis by FTIR Microspectroscopy
Source: Molecules. 2024 Feb 20;29(5):922. doi: 10.3390/molecules29050922 (PMC10934502; doi:10.3390/molecules29050922)
Supplement: Supplementary file 1 [file molecules-29-00922-s001.zip › molecules-2855842-supplementary.pdf]

Cervical Squamous Cell Carcinoma Diagnosis by FTIR Microspectroscopy

Maria M. Félix [1], Mariana V. Tavares [1,2], Inês P. Santos [1], Ana L. M. Batista de Carvalho [1], Luís A. E. Batista de Carvalho [1,*] and Maria Paula M. Marques [1,3]

[1] Molecular Physical-Chemistry R&D Unit, Department of Chemistry, University of Coimbra, 3004-535 Coimbra, Portugal; mmfelix@uc.pt (M.M.F.); mariana.vide.tavares@ipoporto.min-saude.pt (M.V.T.); ips@uc.pt (I.P.S.); pmc@ci.uc.pt (M.P.M.M.)
[2] Gynaecology Department, Portuguese Oncology Institute of Porto, 4200-072 Porto, Portugal
[3] Department of Life Sciences, Faculty of Science and Technology, University of Coimbra, 3000-456 Coimbra, Portugal
* Correspondence: labc@ci.uc.pt; Tel.: +351-239-854-462

Supplementary Information

TABLE OF CONTENTS

1. Figures

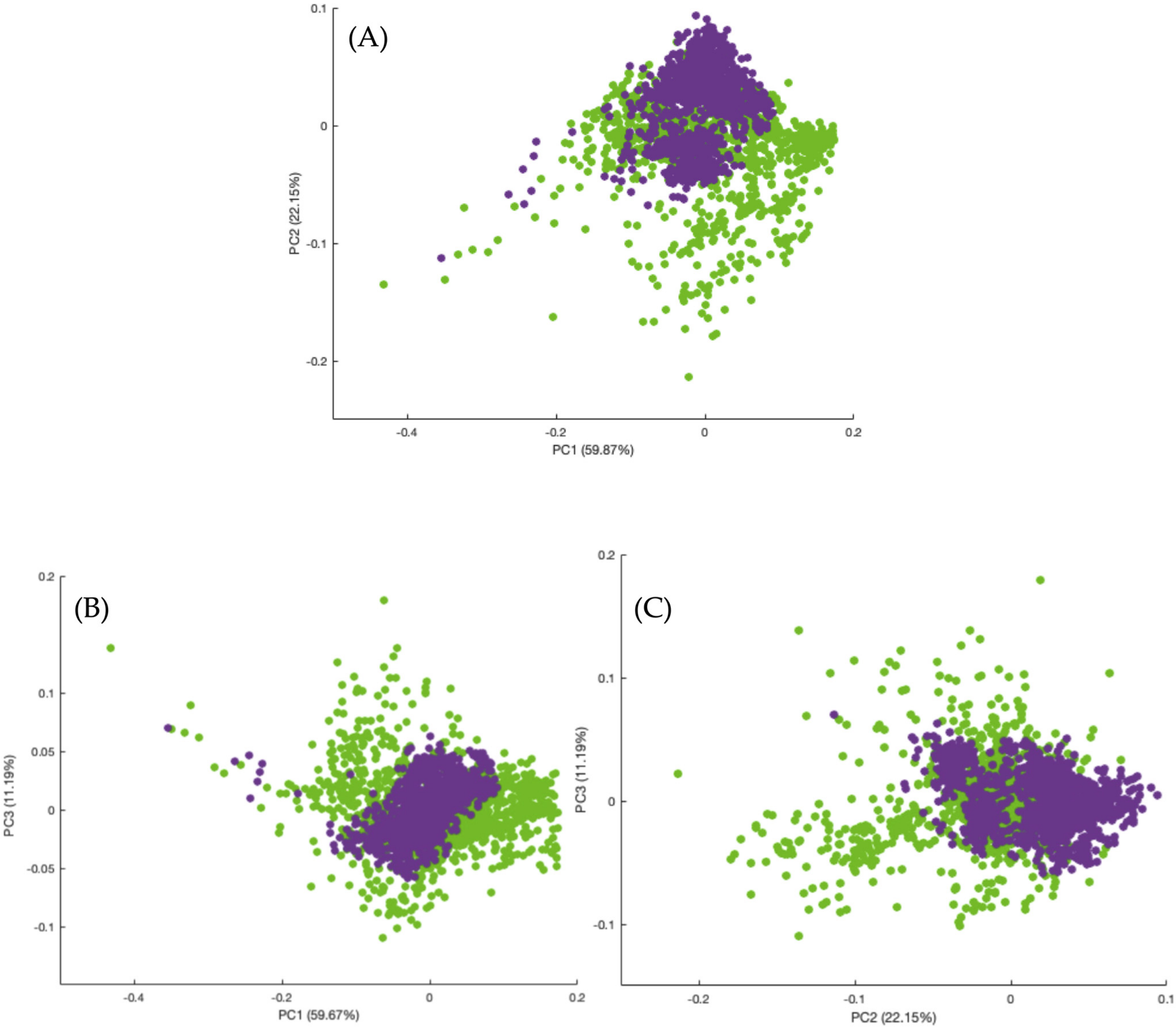

Figure S1. Principal Component Analysis score of FTIR data for cryopreserved normal vs. SCC cervical tissues; **(A)** PC1 *vs.* PC2; **(B)** PC1 *vs.* PC3 and **(C)** PC2 *vs.* PC3
